# Thermal Selection Shifts Genetic Diversity and Performance in Blue Mussel Juveniles

**DOI:** 10.1111/eva.70118

**Published:** 2025-06-19

**Authors:** Jennifer C. Nascimento‐Schulze, Jahangir Vajedsamiei, Tim P. Bean, Lisa Frankholz, Reid S. Brennan, Frank Melzner, Robert P. Ellis

**Affiliations:** ^1^ Biosciences University of Exeter Exeter UK; ^2^ Research Division Marine Ecology GEOMAR Helmholtz Centre for Ocean Research Kiel Kiel Germany; ^3^ The Roslin Institute and Royal (Dick) School of Veterinary Studies The University of Edinburgh Midlothian UK; ^4^ Sustainable Aquaculture Futures University of Exeter Exeter UK

**Keywords:** aquaculture sustainability, Baltic Sea, blue mussel (*Mytilus* spp.), climate change adaptation, experimental selection, genetic adaptation, global warming, SNP‐array genotyping, thermal resilience

## Abstract

Exploring evolutionary and physiological responses to environmental stress is crucial for assessing the effects of climate change on wild populations. Mussels, key inhabitants of the benthos with high ecological and economic value, are a particularly vulnerable species that may be pushed to their ecological limits as warming threatens their survival and population stability. Species within the *Mytilus edulis* complex are commonly found in temperate regions globally; in the Baltic Sea, populations are formed by *M. edulis* and *M. trossulus* hybrids with low levels of *M. galloprovincialis* introgression. This study investigates the mechanisms through which resilience towards global warming may be fast‐tracked in Baltic mussels (Kiel, Germany). For this, we studied two cohorts of juvenile mussels (recently settled animals), one exposed to an extreme heat event early in life and one naïve to this stressor. Both cohorts were later exposed to experimental temperatures ranging from 21°C to 26°C, with animal performance measured after 25 days. Impacts of thermal stress on the genetic composition of each cohort was then assessed by genotyping 50 individuals using a blue mussel 60 K SNP‐array. We observed a significant increase in *M. edulis* genotypes together with a decrease in *M. trossulus* in the challenged cohort, compared to naive juveniles. We also found exposure to high temperature affected performance of mussel cohorts, reducing dry tissue weight of selected individuals. Results from this study provide insights on how selection through thermal stress impacts performance and genetic composition of key globally distributed intertidal species, with important implications for understanding and managing mussel populations under future warming scenarios.

## 1 Introduction

Ongoing climate change is rapidly shifting abiotic and biotic patterns in the ocean, affecting the fitness of marine organisms and the functioning of marine ecosystems worldwide (IPCC 2022). To cope with these rapid environmental changes, marine organisms may migrate to more favourable habitats or exhibit rapid physiological plasticity; however, they ultimately require some degree of rapid genetic adaptation necessary to ensure long‐term population persistence—particularly when migration is not a viable option or in response to extreme events. As these extreme events increasingly push beyond many species' limits, understanding how, and to what extent, species can evolve to tolerate these changes, as well as the broader ecological consequences, is critical for predicting how ocean biodiversity, species distributions, and overall ecosystem structure may be reshaped in the future (Radchuk et al. 2019).

While there is evidence that adaptation can occur in as short as a single generation (Brennan et al. 2019; Griffiths et al. 2021; Campbell‐Staton et al. 2021), we still have a relatively limited understanding of how these selective events act at the genetic level and their phenotypic consequences. Studies combining experimental physiology, genomics, and experimental evolution can provide key insights on the mechanisms underlying rapid selection to extreme conditions (Stillman 2019). Acquisition of new empirical data exploring rapid adaptation in non‐model species and wild populations likely to undergo these shifts is critical to expand our understanding of these processes and the capacity for rapid adaptation across diverse organisms.

Blue mussels are an ideal model to understand the effects of extreme acute selective events. These mussels are a foundation species of the benthos, providing ecosystem services such as nutrient cycling and increased habitat complexity through enhanced spatial structure for associated species (Johannesson et al. 2011; van der Schatte Olivier et al. 2020), and are an important aquaculture and fisheries resource (Avdelas et al. 2021). The three species in the blue mussel *Mytilus* complex native to the northern Atlantic, *M. galloprovincialis* , *M. edulis* , and *M. trossulus*, hybridise in areas of overlapping geographical distribution (Fraïsse et al. 2016). In the Baltic Sea, blue mussel populations are a highly introgressed swarm composed of all three species (Vendrami et al. 2020). In this region, blue mussels are locally adapted to the natural salinity gradient (Knöbel et al. 2021) transitioning from fully marine in the west to nearly freshwater at the eastern portion of the basin (Meier et al. 2006), with genotype distribution aligned with this gradual change. These populations already cope with environmental pressures, such as warming, at levels that most coastal areas are not expected to experience until the end of the century (Reusch et al. 2018). However, little is known concerning the implications of thermal stress in these populations on a genomic level.

The Baltic Sea can serve as a model to understand population responses to global change as it is experiencing environmental shifts at a faster rate than most other regions (Reusch et al. 2018). It is one of the fastest warming seas globally (Belkin 2009) and surface temperatures (SST) have increased by more than 0.3°C over the past decades, with warming trends being especially pronounced during summer months compared to winter (Meier et al. 2022). For instance, in 2018, the warmest summer since 1990 (Naumann et al. 2018), heatwaves raised temperatures by more than 4°C above the 28‐year mean (1990–2018) in the southern portion of the basin (Meier et al. 2022). Shifting temperature regimes, either through modified mean seasonal temperatures or isolated shorter extreme events, can contribute to community shifts and set off a chain reaction of biodiversity loss, pushing this ecosystem towards an alternate state (Johannesson et al. 2011; Pansch et al. 2018; Reusch et al. 2005).

Temperature is a key regulator of ectotherm physiology (Somero et al. 2017). Mussels respond to acute heat by increasing their metabolism, reducing high energy demanding processes (e.g., growth) and/or supplementing with anaerobic metabolism to meet rising energy demands (Anestis et al. 2007; Braby and Somero 2006; Tagliarolo and McQuaid 2015; Vajedsamiei, Wahl, et al. 2021; Zittier et al. 2015). Whilst these strategies can guarantee short‐term survival in non‐optimal temperatures, prolonged exposure beyond CT_max_ causes a metabolic supply–demand mismatch, impairing performance and leading to mortality (Pörtner and Farrell 2008; Ritchie 2018; Anestis et al. 2007; Feidantsis et al. 2020). Indeed, mass mortality events have been reported in Mediterranean mussel beds following marine heatwaves (Bracchetti et al. 2024).

The capacity to adjust metabolic rates in response to temperature is a critical survival trait and varies across mussel species (Pörtner and Farrell 2008; Vajedsamiei, Wahl, et al. 2021). For example, the upper thermal limits for maintaining a positive Scope for Growth (i.e., the extra energy available for growth after meeting basic survival needs) in *M. trossulus* , *M. edulis* , and *M. galloprovincialis* acclimated to summer temperatures from the northwestern Pacific and northeastern Atlantic coasts are ca. 17°C, 23°C, and 30°C, respectively (Fly and Hilbish 2013). This suggests that at elevated temperatures, *M. trossulus* genotypes have a lower performance in comparison to the other two species, with likely implications for growth, survival, and reproductive output. The extent to which different *Mytilus* species genotypes within hybrid populations influence responses to thermal stress remains unclear.

**FIGURE 1:**
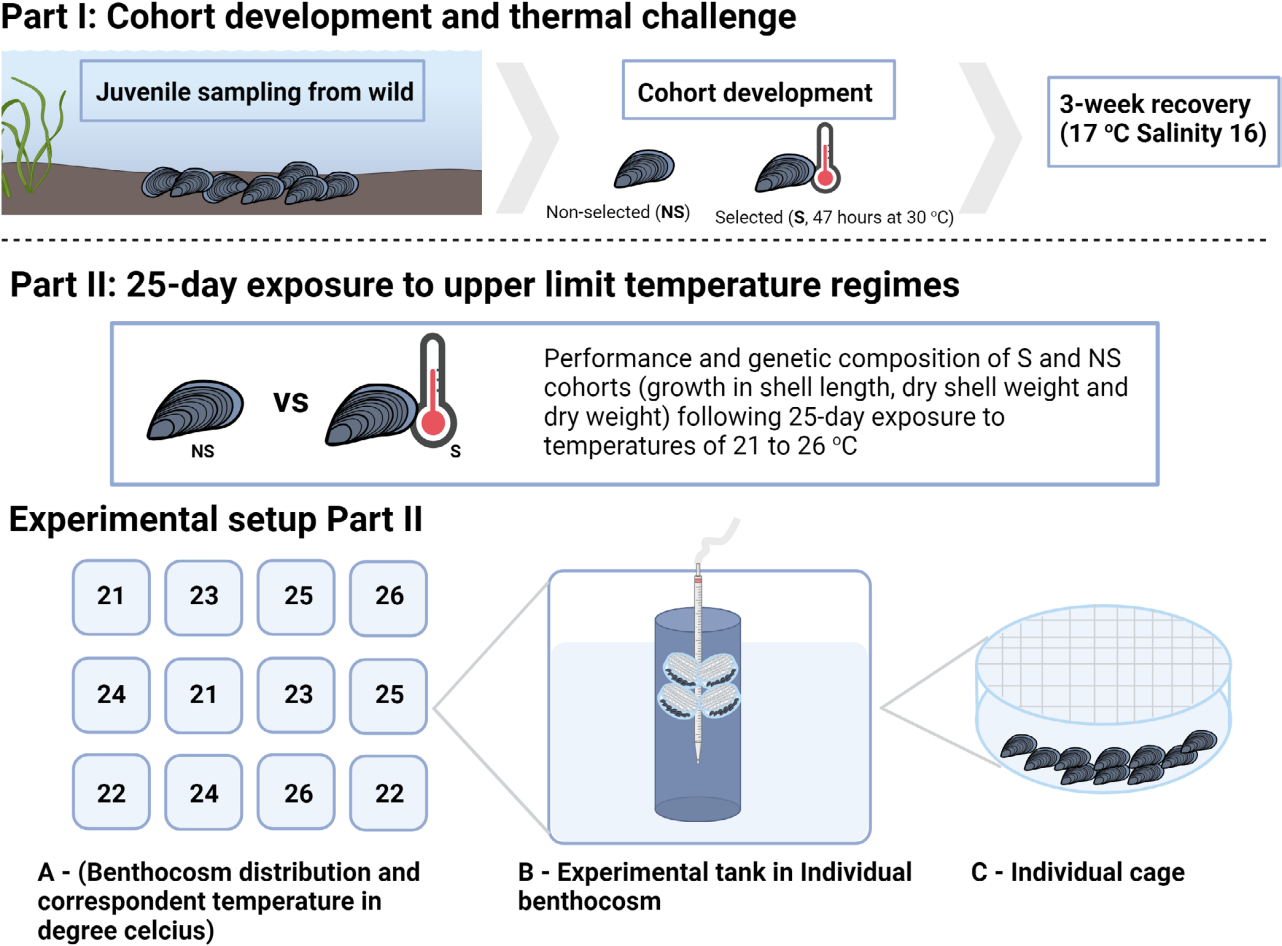
Overview of the experiment specifying the main steps. Part I describes the development of both cohorts (selected and non‐selected) following selection after a simulated heatwave event. Part II outlines the experimental set‐up for the 25‐day thermal challenge conducted in a controlled indoor benthocosm system, with a representation of the benthocosm layout (A), a schematic representation of the tank setup within individual benthocosms; experimental cylinders filled with seawater, aerated via a plastic pipette, and holding four chambers containing mussels (B); and finally an individual mussel chamber (C).

In this study, we addressed this knowledge gap via a two‐step experimental approach (Figure 1). First, to assess the implications of thermal stress on the genetic makeup of mussel populations, we collected spat (i.e., recently settled juveniles) from a single settlement event within Kiel Fjord and developed two distinct cohorts, one selected for thermal tolerance via an acute but intense exposure to a thermal stress event (30°C for 47 h), and one naïve to this stressor. Individuals from both cohorts were genotyped with the 60 K Blue mussel SNP‐array (Nascimento‐Schulze et al. 2023) a genomic tool that enables consistent and cost‐effective genotyping of individuals across thousands of predefined loci, ensuring reproducibility of analysis (Robledo et al. 2018). We hypothesised that temperature selection would create a genetically distinct cohort, favouring genotypes more resilient to higher temperatures. Specifically, we predicted that the fraction of *M. trossulus* alleles would decrease following temperature selection. Second, we tested the impacts of post‐selection thermal stress on mussel performance. For this, we exposed individuals from both developed cohorts to constant Baltic summer temperature scenarios, ranging from current to forecasted end‐of‐century summertime extremes, over a 25‐day period, and assessed whole organism performance in shell growth and dry tissue gain. We hypothesised that heat‐selected lines would outperform non‐selected lines at higher temperatures.

## 2 Materials and Methods

### 2.1 Development of Selected and Non‐Selected Juvenile Cohorts

#### 2.1.1 Selection Event

Approximately 500,000 mussel spat (i.e., settled individuals) were collected by hand from subtidal Kiel Fjord (54.328588, 10.148210, Baltic Sea, Germany) on July 28th, 2021, from approximately 0.5 m depth, and gently sorted using a 1‐mm mesh size plastic sieve suspended in a 10 L bucket of seawater. On the following day, around 50,000 spat were evenly distributed into five separate 10 L tanks (~ 10,000 spat per tank) and kept at 17°C (temperature‐controlled room at GEOMAR) with 16 PSU natural seawater filtered through a 0.5 μm mesh. For the next 8 days, husbandry practices involved 50% water exchanges daily and the addition of 100 mL of *Rhodomonas salina* (~1–2 × 10^6^ cells mL^−1^) as food. The remaining 450,000 spat were thermally challenged: first, being evenly distributed among 16 × 2 L tanks (ca. 25,000 per tank) filled with 600 mL of 16 PSU natural seawater, filtered with a 0.5 μm mesh, and exposed for 30 min to a non‐lethal elevated temperature (26.5°C), with constant aeration supplied through 5 mL plastic pipette tips. The experimental tanks were placed into four steel water baths that maintained target temperatures with a deviation of less than 0.1°C (model Haake SWB25, Thermo Scientific). A further 1 L of filtered sea water (FSW; filtered across a series of filters 10 and 1 μm mesh) at 30°C was added to each tank, water baths were set to 30°C and target temperatures were reached within ~ 15 min. Spat were then kept at this temperature for 47 h.

Three days post collection, all heat‐treated spat (dead or alive) were washed in FSW to remove tissue of dead mussels and then transferred from the 16 × 2 L tanks into 4 × 10 L tanks with FSW (salinity 16 PSU, temperature 17°C ± 0.2°C, constant aeration), located adjacent to the tanks housing the naïve animals in a temperature‐controlled room. For the first 2 days post‐transfer, water changes (~ 95% of volume) were undertaken daily to remove the organic remains of dead animals, followed by the addition of 200 mL of *R. salina* (~ 1–2 × 10^6^ cells mL^−1^) to each tank. Over the following 9 days, husbandry practices shifted to daily 50% water exchange and the addition of 100 mL of *R. salina* (~ 1–2 × 10^6^ cells mL^−1^). Heat‐selected spat were then stained with calcein green and naïve animals with calcein blue (50 mg L^−1^ on day 1, and 25 mg L^−1^ on days 2–9), added immediately after the water changes to fluorescently label live individuals. Both chemicals are non‐toxic to mussels.

At 14‐days post collection, heat‐selected spat from the four tanks were mixed and three 500 μL sub‐samples of mussels were pipetted into a 0.5 mL Eppendorf tube, with survival rates assessed. Due to the calcein shell labelling procedure, living spat appeared bright green under the stereomicroscope equipped with standard GFP‐filter sets (Leica M165 FC), and 600 living spat were found and kept for the subsequent steps. In addition, approximately 600 spat were randomly sorted from the ~ 50,000 naive spat kept in the five maintenance tanks at constant temperatures of 17°C. No mortalities were observed in the naive group. Each group of 600 naïve or selected spat was then equally distributed among four 50 mL containers (150 individuals in each container, four containers per spat line), which were closed with mesh (300 μm). Two of these containers, one containing selected and one containing the non‐selected spat were kept in one 10 L aquarium (‘conditioning tank’, total of four tanks) for 7 days at 17°C and 16 PSU, fed every day with an initial concentration of ca. 8000 *R. salina* cells mL^−1^ to ensure comparable condition between spat from the two different treatment groups for the following experiments.

In the following sections of this manuscript, spat surviving the thermal stress exposure are referred to as ‘selected’ (S), whilst spat not exposed to thermal stress are termed ‘non‐selected’ (NS). These spat were exposed to a thermal challenge in the Kiel Indoor Benthocosm facilities (KIB, Pansch and Hiebenthal 2019).

#### 2.1.2 Second Stress Exposure

Following the initial development of selected and non‐selected spat cohorts, 960 individuals (480 selected and 480 non‐selected) were randomly selected for a follow‐up experiment, in which we assessed the impact of thermal stress on juvenile performance. Beginning on August 20th, spat were exposed to five different temperature treatments (21°C, 22°C, 23°C, 24°C, 25°C and 26°C) over a 25‐day period. The 26°C treatment represented a predicted end‐of‐century peak daily marine heatwave temperature in the study region, sub‐lethal to mussels for periods of < 1 month. The 21°C treatment represents a present‐day maximum summer temperature, which is in the optimal range for mussel growth. The intermediate temperatures within the experimental range were selected to identify any subtle, but significant, alterations in thermal performance or thermal breakpoints in larval mussel performance in this study. The experiment was conducted using the KIB. In this facility, 12 × 600 L polyethylene tanks (i.e., benthocosms) equipped with GHL Profilux computers and thermal sensors (GHL GmbH), controlling heaters and chillers (Aquamedic), enabled the maintenance and recording of water temperature. Two experimental cylinders were assigned to each of six experimental temperatures. KIB tanks were used as water baths and housed the 12 animal incubation cylinders (two per water bath). Animals were placed into plastic cages (30 mm O.D, approximately 10 mm length) sealed on the top and the bottom with 300 μm nylon mesh (Figure 1). Experimental cages were placed into 14 L plastic cylindrical tanks, allowing water flow and food uptake to occur. Twenty juveniles were kept in each cage, with each treatment (S and NS) having two replicate cages per experimental tank. Thus, a total of 80 S and 80 NS animals were incubated at each temperature. Cages were tied to 15 mL pipettes that were used for aeration at the same water column height, providing similar environmental conditions between replicates of each treatment (Figure 1).

Initial shell length was measured in a sub‐sample of 60 selected and 60 non‐selected individuals, which were removed from the conditioning tank before being distributed into experimental cages on day 0, 1 day prior to the start of the 25‐day thermal exposure experiment. The impact of elevated temperature on mussel spat was assessed by measuring larval performance via growth as shell length, dry tissue weight, and dry shell weight on day 25, at the end of the challenge, from at least 10 animals per cage. Raw data values in the results section are presented as mean ± standard deviation.

#### 2.1.3 Husbandry

Throughout the duration of this experiment, spat were fed with monocultures of *Rhodomonas salina*, added at a concentration of ~8000 cells mL^−1^ (Riisgård et al., 2011) to the experimental cylinders after every water exchange. Before the start of the main experiment, we measured *R. Salinas* concentration daily over 72 h in all experimental tanks set to their respective temperature, to guarantee that drops in cell concentration were caused by food consumption rather than algae death (Table S1). Cell concentration was measured daily (particle size 5–8 um) using a Coulter counter (Coulter Z2, Beckman Coulter GmbH), in all experimental tanks. If *R. salina* concentration in any of the experimental tanks dropped to threshold values in the range of 1000 cells mL^−1^, full water exchanges were performed in all the tanks in order to ensure that *R. salina* was constantly available in the water column at optimal concentrations. Water changes were conducted with FSW every 3rd day (days 1–7), every other day (days 8–20), and daily after day 21 (Table S2).

#### 2.1.4 Growth, Dry Body Mass and Shell Mass Assessment

Shell length was measured using vernier callipers. Dry tissue weight and shell weight were measured as previously described. Briefly, sampled spat were killed in a microwave (400 watts, 30 s), and their soft tissue was removed from their shell under a stereomicroscope. Shell and soft tissues were separately placed individually into pre‐weighed tin foil boats dried at 80°C, for a minimum of 72 h, and reweighed to determine the dry tissue weight and shell weight (mg) by subtracting the value of the empty foil boat from the foil boat containing shell or soft tissue. Raw data values in the results section are presented as mean ± standard deviation.

#### 2.1.5 Assessing Structure and Genomic Relatedness of the Two Cohorts

The genetic composition of selected and non‐selected cohorts was analysed using 50 spat from each treatment, collected on day 0 of the second experiment, i.e., the day prior to the start of the 25‐day exposure challenge, with sampled spat preserved in 90% ethanol. Samples were sent to Identigen Ltd. for DNA extraction and sequencing, using the 60 K blue mussel SNP‐array (Nascimento‐Schulze et al. 2023).

To analyse the resulting sequencing data, a quality control filter for marker call rate (CR) of > 95% and sample CR > 90% was applied using PLINK v1.9. We applied a minor allele frequency (MAF) filter < 0.01. A principal component analysis (PCA) was applied to genotypes generated with the array, pruning out putatively linked loci using a window size of 50 Kb, a step size of 10 Kb and an *r* ^2^ threshold of 0.1 with PLINK v1.7 (Purcell et al. 2007). We explored introgression in spat by applying an admixture analysis (admixture v1.3; Alexander and Lange 2011). In this analysis, the best fitting number of clusters representing the ancestry of a population is estimated where *k* has the lowest cross‐validation error value. For this study, we tested *k* values between 2 and 10. In order to allocate a species to the ancestry cluster generated in this first admixture analysis, we ran an additional admixture analysis including 5 additional populations, Kiel (Baltic Germany, GK), Ahrenshoop (Baltic Germany, GA), Finland (Baltic Finland, FIN), Bude (North Devon coast of the UK) and Budleigh (South Devon coast of the UK). These populations have been genotyped with the blue mussel multi‐species 60 K array in previous studies (Nascimento‐Schulze et al. 2023; Nascimento‐Schulze et al. unpublished data), and their ancestry is known to be *M. edulis/M. trossulus* hybrids for GK and GA and FIN populations, with an increased frequency of *M. trossulus* genotypes in FIN, *M. galloprovincialis* ancestry dominating Bude and *M. edulis* prevailing in Budleigh. Admixture results of this data set are presented in Tables S3 and S4, as well as visualised in Figures S1 and S2.

#### 2.1.6 Statistical Analysis

##### 2.1.6.1 Spat Performance

Shell length data collected from individuals at day 0, prior to the start of the 25‐day thermal challenge, was first tested for normality and homogeneity of variance using Shapiro–Wilk and Levene's tests respectively before a one‐way ANOVA analysis was applied to test for the effects of temperature selection.

We investigated the effects of temperature on the dry tissue weight, shell weight and shell length of selected and non‐selected individuals following the 25‐day thermal challenge. These effects were modelled as generalised additive mixed models (GAMMs) using the *gam* function from the *mgcv* package. In the GAMMs, *temperature* was defined as a smooth‐effect predictor (with linear and/or nonlinear effects) and *selection* as an ordered factor. To test the effects of temperature within and between the selected and non‐selected groups, temperature was modelled as a smoother within each level of the selection group (selected vs. non‐selected). More specifically, we used t‐statistics and f‐statistics (Wald test), the intercept (or the mean) and the slope or nonlinearity (effective degrees of freedom, *edf*) of the reference level smoother (*non‐selected*), with each compared to zero, respectively; and the treatment level (*selected*) smoother's estimates were then compared to those of the reference level. In addition, the random effects of *water bath* and *cage*, as possible causes of residual dependence, were included, and for unbiased estimation of variance components, GAMMs were fitted using restricted maximum likelihood.

The models were first fitted with the *identity* link function with an assumption of residuals having a gaussian distribution around the predicted means. The assumptions regarding the distributions of residuals were checked via the *DHARMa* package. As it was determined that the residual assumptions were violated, we fitted the models assuming the scaled t‐distribution (*scat*) of residuals and found this assumption to be valid. For shell length, the *log*_*10*_ link function was also needed to validate the residual assumption. In terms of the Akaike Information Criterion (AIC), the *scat* models were also superior to the gaussian models. We used the *predict* function from *car* package to predict the means and confidence intervals of responses to predictors. A *p* value of < 0.05 was assumed as the significance level for all analyses. All analyses were conducted in R v4.3.1. Raw data values in the results section are presented as mean ± standard deviation.

##### 2.1.6.2 Population Genetic Composition

We compared the genetic composition of the two mussel cohorts (selected and non‐selected) to investigate potential differences in ancestry proportions resulting from the selection event. For this, we specified a Bayesian regression model assuming a Logistic‐Normal distribution for the ancestry proportions, utilising the *brms* package (Bürkner 2017) in R v4.3.1. The Bayesian Logistic‐Normal Model was chosen as it allows for the comparison of mean proportions and variability within the compositional data, while accounting for correlations between components, making it a robust choice for our analysis. The model was fitted using the No‐U‐Turn Sampler (NUTS) within the Hamiltonian Monte Carlo framework. A thorough description of the models tested and applied is available in the Supplementary Information, Part II; Figures S4–S6).

## 3 Results

### 3.1 Impacts of Increased Temperature on Cohort Survival and Genetic Makeup

At 10 days post thermal challenge, the survival rate of the selected spat cohort was approximately 8%. No deaths were observed in non‐selected mussels during this period.

From the 15,049 genotyped loci with a CR > 95%, a total of 11,288 loci were retained after applying the MAF > 0.01. After variant pruning, a total of 10,085 putatively non‐linked loci were used for PCA and Admixture analysis.

**FIGURE 2:**
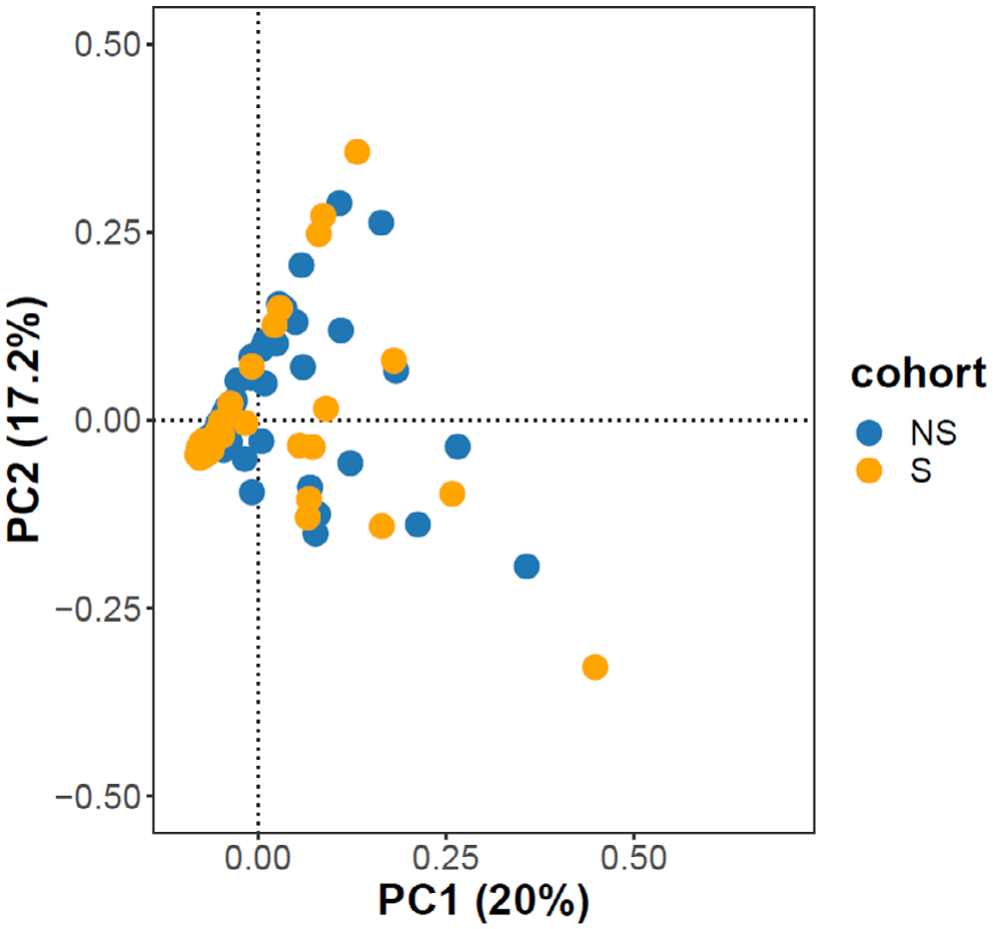
Scatter plots of individual variation in PC 1 and 2 scores resulting from PCA applied to the blue mussel juveniles from Kiel fjord, Germany. Selected (S) and non‐selected (NS) individuals were genotyped with the blue mussel multi‐species 60 K SNP array. The proportion of overall variation explained by each PC are given in percentages.

The PCA analysis revealed no obvious stratification between selected and non‐selected cohorts (Figure 2). In this analysis, PC1 explained 20% of the variance, while PC2 and PC3 explained 17.2% and 7.64%, respectively.

**FIGURE 3:**
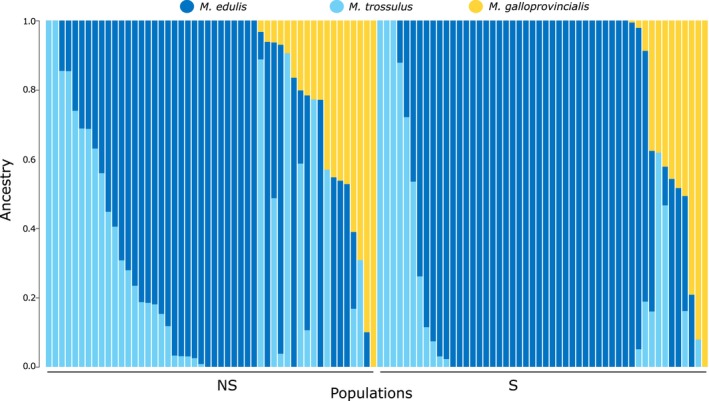
Results of genetic admixture analysis and ancestry inference in the selected (S) and non‐selected (NS) populations. Cluster membership coefficients (Q) where each individual is represented by a column partitioned into segments of different colour, the length of which indicates the posterior probability of membership in each cluster. Solid bars represent an individual from a single species background.

The best fitting number of ancestry clusters in the admixture analysis was three (Table S4). Results from the admixture coefficients (Q) inferring the three clusters are presented in Figure 3. Based on the analysis, including the control populations (Figures S1, S2), we could infer the species for each of the three clusters present in the S and NS cohorts, with the dark‐blue cluster representing *M. edulis* ancestry, light‐blue *M. trossulus* , and yellow *M. galloprovincialis* . In this analysis, we observe that most of the genotypes are allocated to *M. edulis* , followed by *M. trossulus* and finally *M. galloprovincialis* .

The genetic composition of selected vs. non‐selected populations revealed different trends for *M. edulis* and *M. galloprovincialis* . Raw proportions from the admixture analysis show that the non‐selected population was composed of *M. edulis* (0.58), *M. trossulus* (0.29), and *M. galloprovincialis* (0.13), whereas the selected population had proportions of *M. edulis* (0.74), *M. trossulus* (0.15), and *M. galloprovincialis* (0.11). Bayesian mixed‐effects modelling on the logit scale showed strong evidence for an increase in the proportion of *M. edulis* in the selected population compared to the non‐selected population, with an estimated non‐selected intercept of 2.40 (95% CI: 0.38 to 4.38), and a selected population effect 3.75 (95% CI: 0.74 to 6.42) (Supporting Information, Part II). The increase in *M. edulis* genotypes comes at the expense of *M. trossulus*, as although the model indicated a positive trend towards an increased proportion of *M. galloprovincialis* in the selected population, the credible interval included negative values and was therefore not significant (1.93, 95% CI: −0.74 to 4.62) (Supporting Information, Part II), thus the significant increase in *M. edulis* is due to a reciprocal significant decrease in *M. trossulus* . The family‐specific parameters showed significant variability in the proportions of both *M. edulis* (*σ*q2 = 7.24, 95% CI: 6.32–8.33) and *M. galloprovincialis* (*σ*q3 = 6.73, 95% CI: 5.87–7.74) across individuals, pointing to considerable heterogeneity within the populations. Additionally, the model found a moderate positive correlation between and *M. edulis* and *M. galloprovincialis* ancestry (correlation = 0.48, 95% CI: 0.33–0.62), suggesting that increases in proportion of *M. edulis* ancestry within the population tended to be accompanied by increases in *M. galloprovincialis* ancestry.

### 3.2 Impacts of Increased Temperature on Shell Length, Dry Tissue Weight, and Shell Mass

No significant difference in shell length was observed between selected (2.02 ± 0.33 mm) and non‐selected (1.99 ± 0.32 mm) cohorts at day‐0, prior to the start of the 25‐day thermal challenge (Figure S3, Table S5, *p* > 0.05).

**FIGURE 4:**
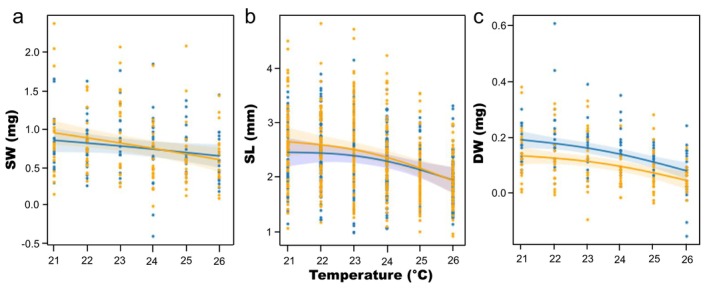
(a) Shell weight (SW), (b) shell length (SL) and (c) dry tissue weight (DW) of selected (S, orange) and non‐selected (NS, blue) spat across experimental temperatures (21°C–26°C), after 25 days of exposure to different temperatures. Each of the data points represents the measurement of an individual spat. These relations were modelled as additive mixed models assuming scaled t‐distributions of residuals. Solid lines represent predicted means and shades represent 95% confidence intervals.

Exposing spat to different experimental temperatures over a 25‐day period did not result in a significant difference in shell weight of individuals (Figure 4a, Table 1), with shell weight of selected spat being 0.79 ± 0.5 mg, while non‐selected spat were 0.77 ± 0.36 mg.

**TABLE 1:** Main and interactive effects of elevated temperature and selection on shell weight (SW), shell length (SL) or dry tissue weight (DW) of *Mytilus* spat following a 25‐day challenge.

SW 25‐days	Estimate	Std. Error	*t*	*p*
A. Parametric coefficients				
Intercept (non‐selected)	0.7492	0.0392	19.0895	< 0.0001
Intercept (selected)	−0.0315	0.0510	−0.6189	0.5360

SW 25‐days	edf	Ref.df	*F*	*p*
B. Smooth terms				
s(temperature, non‐selected)	1.4395	1.6167	2.0979	0.1726
s(temperature, selected)	1.0001	1.0002	0.6174	0.4320
s(cage)	6.0661	22.0000	8.6526	0.0832

SL 25‐days	Estimate	Std. Error	*t*	*p*
A. Parametric coefficients				
Intercept (non‐selected)	0.3777	0.0081	46.3824	< 0.0001
Intercept (selected)	0.0126	0.0068	1.8364	0.0663

SL 25‐days	edf	Ref.df	*F*	*p*
B. Smooth terms				
s(temperature, non‐selected)	1.7442	1.7886	13.3916	0.0006
s(temperature, selected)	1.0005	1.0010	2.3425	0.1260
s(cage)	16.8297	23.0000	80.1398	< 0.0001

DW 25‐days	Estimate	Std. Error	*t*	*p*
A. Parametric coefficients				
Intercept (non‐selected)	0.1418	0.0087	16.3570	< 0.0001
Intercept (selected)	−0.0459	0.0115	−3.9794	0.0001

DW 25‐days	edf	Ref.df	*F*	*p*
B. Smooth terms				
s(temperature, non‐selected)	1.6596	1.8153	9.6515	0.0001
s(temperature, selected)	1.3257	1.5142	0.2626	0.5926
s(cage)	4.6894	22.0000	0.2925	0.1261

No significant impact of temperature or selection was observed on shell length between both cohorts (Figure 4b, Table 1, *p* > 0.05). The mean shell length of selected and non‐selected cohorts at day 25 was 2.33 ± 0.52 mm and 2.47 ± 0.67 mm, respectively, suggesting a marginal non‐significant (*p* = 0.0663) effect of selection on subsequent growth. The smooth effect of temperature was significant overall, as shown by the non‐selected cohort (*p* = 0.0006), but was not significantly different between the both cohorts (*p* = 0.1260), with non‐selected juveniles at 26°C smaller (2.04 mm ± 0.45) than those at 21°C (2.37 ± 0.49 mm).

For dry tissue weight analysis, the intercept for the selected cohort, representing its difference from the non‐selected baseline, was significantly lower (*p* = 0.0001; Figure 4c, Table 1). These results indicate an effect of prior selection on dry tissue mass in all temperature treatments. The smooth effect of temperature was significant overall, as shown by the non‐selected group (*p* = 0.0001), but was not significantly different between the selected and non‐selected groups (*p* = 0.5926). Observed dry tissue weight of non‐selected spat decreased from 0.16 ± 0.06 mg at 21°C to 0.06 ± 0.10 mg at 26°C, and dry tissue weight of selected spat decreased from 0.14 ± 0.11 mg at 21°C to 0.05 ± 0.15 mg at 26°C.

These relations were modelled as additive mixed models assuming scaled t‐distributions of residuals. The intercept (or the mean) and the slope or nonlinearity (effective degrees of freedom, edf) of the reference level smoother (naïve) were compared to zero, respectively; and, the treatment level (selected) smoother's estimates were then compared to the reference level. In addition, the random intercept effects of cage were tested. Significant impacts were considered where *p*‐value ≤ 0.05.

## 4 Discussion

In this study, we assessed the impact of strongly elevated summer temperatures on the genetic variation and performance of Baltic juvenile *Mytilus* spp. We found that selection to strongly increased temperatures resulted in an enrichment of *M. edulis* dominated genotypes and concurrently led to reduced tissue growth, in a follow‐up growth trial at all tested temperatures. We therefore provide the first evidence that temperature could play an important role in the genomic and phenotypic composition of *Mytilus* mussels in the Baltic Sea. This trend was evident after only a single generation and selection event, and may be indicative of processes that occur in the wild during warm summers. In addition, this rapid response to selection suggests that *Mytilus spp* may be amenable to artificial selection to shift genomic composition and physiological traits of populations, increasing their resilience to ever more extreme global change. More work is needed to understand how genotypes respond to selection in the wild, and to demonstrate if the development of thermally resilient populations via selection in the lab over multiple generations is feasible. However, our results are an important initial step to show that this additional work is necessary and promising.

### 4.1 Impacts of Increased Temperature on the Genetic Variation of Mussel Spat

We investigated whether exposure to thermal stress can modify the genetic structure of juvenile blue mussel populations by comparing the genetic composition of two spat cohorts, before and after selection, using a multi‐species 60 K SNP‐array (Nascimento‐Schulze et al. 2023). The genetic background of the population generally agrees with previously published data on mussels originating from the Baltic Sea, where three species contribute to the population: *M. edulis* , *M. trossulus* and *M. galloprovincialis* . Nonetheless, the contribution of *M. galloprovincialis* ancestry in spat analysed in this study is considerably higher than has been previously described in this region. While Vendrami et al. (2020) genotyped adult mussels, juveniles (approximately 2 months old) were genotyped in our study. The Baltic Sea is a unique environment due to its salinity gradient and limited water mixing with the North Atlantic through the Danish Straight. Individuals possessing *M. galloprovincialis* alleles may be sporadically transported into this region by water currents, carried by ballast water, or attached to other recreational vessels. It is possible that differential survival of genotypes through development could explain the different ancestries. However, more work would be needed to verify this hypothesis.

We find evidence that temperature selection shifted the genetic composition of blue mussels, with a higher frequency of *M. edulis* genotypes in the selected cohort in comparison to the non‐selected population. Genotype‐dependent mortality has been previously observed in mussels (Han et al. 2020; Koehn et al. 1980), supporting the rapid responses to selection in our study. For example, post‐settlement selection acting on the *Lap* allele has been observed in *M. edulis* inhabiting a salinity gradient, with the presence of the allele decreasing through development in low salinities (Koehn et al. 1980). SSTs found across the natural distribution of *M. edulis* and *M. galloprovincialis* are higher than those found within the range of *M. trossulus* (Table S6; Popovic and Riginos 2020). These observations support the decreasing proportion of *M. trossulus* alleles in the selected population in our study as being driven by selection to temperature‐tolerant genotypes. Local adaptation to low salinity may have favoured the increase of *M. edulis* genotypes rather than *M. galloprovincialis* in the selected population. Nonetheless, despite showing a significant shift in the population genotype between the non‐selected and selected populations, the uncertainty from the Bayesian modeling indicates slight caution should be taken when interpreting this result, due to the genomic heterogeneity in ancestry at an individual level, and thus the uncertainty of the overall magnitude of this shift. Moreover, further research is required to understand how changes in the genomic composition of spat populations manifest in the adult population.

On a genomic level, rapid adaptation following a single generation selection event has been previously demonstrated in multiple invertebrate species, including to salinity in larvae of a closely related oyster species (Griffiths et al. 2021), as well as to ocean acidification in mussels (Bitter et al. 2019) and sea‐urchin larvae (Brennan et al. 2019). Nonetheless, this is the first study to investigate rapid adaptation of population genotype following a single generation selection event in a study system comprising individuals with admixed ancestry. Thus, our results provide critical advances in our understanding of species interactions and introgression in a hybrid zone under changing climatic conditions. To fully understand the impact of this selection on population composition, however, it is key that future work determines whether this effect persists across time and generations, or whether temporally balanced selection may reduce the impact of this event at a population level (e.g., Durland et al. 2021). In addition, it is critical to assess the effects of environmental stress across all life stages: while our study focused on juveniles, selection pressures may vary substantially between life stages. Investigating these differences will offer a more comprehensive understanding of how rapid selection shapes the evolutionary trajectory of these organisms.

### 4.2 Impacts of Increased Temperature on Dry Tissue Weight, Shell Length, and Shell Mass

Our study found that after 25 days of exposure to elevated temperatures, selected spat had a lower dry tissue weight compared to non‐selected individuals. In addition, shell length of non‐selected individuals decreased with increasing temperature.

The pervasive impacts of temperature, from molecular to whole‐organism levels, affect performance and define the thermal window of a population. Studies on *Mytilus galloprovincialis* show that intermediate warming of 24°C–26°C, shifts pyruvate kinase to a less active form of the enzyme, leading to hypometabolism and subsequently to metabolic suppression (Anestis et al. 2007). This metabolic suppression enables individuals to lower their energy requirements and allocate surplus energy to the maintenance of basic functions in warm environments (Schulte et al. 2011), and has been observed in Baltic mussels in temperatures beyond the population's threshold (Vajedsamiei, Melzner, et al. 2021; Vajedsamiei, Wahl, et al. 2021). Therefore, metabolic suppression may have potentially enabled energy to be allocated from growth to physiological processes required to counteract the extreme temperatures. This energetic trade‐off may have contributed to the survival of the selected cohort during the thermal selection event, but likely at the expense of reduced tissue growth in selected individuals.

The higher experimental temperatures, 23°C–26°C, encapsulate conditions the mussel population will face during the most extreme warm summer periods, from the present day through to the end of the century (Meier et al. 2022; Pansch et al. 2018; Vajedsamiei, Melzner, et al. 2021; Vajedsamiei, Wahl, et al. 2021). Juvenile mussels will unavoidably experience elevated SST and extreme weather events during their development in the Baltic Sea. Our results suggest that these temperatures can exceed the thermal optima for these juvenile mussels. Nonetheless, to confirm whether selection has favoured individuals with lower metabolic rates, oxygen consumption, feeding and excretion rates must be assessed in a follow‐up study. Furthermore, to assess whether reduced dry tissue weight demonstrates impaired organism function, the persistence of this phenomenon across to adult organisms must also be investigated.

## 5 Conclusions and Future Perspectives

Climate change is projected to modify oceanic physiochemical parameters globally, which have been relatively stable for millennia, with implications for marine ecosystems. Blue mussels are a species group of high economic and ecological value; thus, guaranteeing that both natural and commercial stocks can thrive under new environmental conditions is a matter of critical importance.

This is the first study to characterise in depth the genetic composition of spat from the Southern coast of the Baltic Sea, using a genomic approach. Using a single generational selection approach to elevated temperature, we observed a clear increase in dominance of *M. edulis* ancestry in the selected population. Our study shows that selection impacts spat performance, with selected individuals able to maintain shell growth at a comparable rate to NS individuals, but at the expense of maintenance of somatic tissue growth. Furthermore, the higher resilience and the fast response to selection of *M. edulis* genotypes to elevated temperature suggest that alleles from this species may confer resilience to increasing temperatures caused by global change, even in highly admixed populations. The differences in the performance and the rapid shift in genetic composition between selected and non‐selected cohorts in our study indicate an important role of temperature in this species complex that justifies further investigation. Such information will advance our understanding and ability to quantify the response of blue mussels to thermal stress, as well as their ability to acclimate and/or adapt to such a stressor, in a future ocean. Combining the use of a blue mussel genomic toolbox with knowledge of species‐specific physiological mechanisms can ultimately contribute to the development of thermally resilient cohorts and is a key avenue for further research.

## Conflicts of Interest

The authors declare no conflicts of interest.

## Supporting information


Data S1.

## Data Availability

Raw CEL files are available on GEOME, under the MytiSNP team environment, and can be accessed using the following ARK ID link https://n2t.net/ark:/21547/R2602. Code to run all analysis and produce all figures, and raw admixture output data can be found on Github: https://doi.org/10.5281/zenodo.14362983.
